# COVID-19 Vaccination Refusal—Which Factors are Related in the Czech Republic, One of the Most Affected Countries in the World?

**DOI:** 10.3389/ijph.2023.1605375

**Published:** 2023-03-14

**Authors:** Radka Zidkova, Klara Malinakova, Jitse P. van Dijk, Peter Tavel

**Affiliations:** ^1^ Olomouc University Social Health Institute, Palacký University Olomouc, Olomouc, Czechia; ^2^ Department of Community and Occupational Medicine, University Medical Center Groningen, University of Groningen, Groningen, Netherlands; ^3^ Graduate School Kosice Institute for Society and Health, P.J. Safarik University in Kosice, Kosice, Slovakia

**Keywords:** COVID-19, vaccination, knowledge, government trust, vaccine refusal, personal characteristic

## Abstract

**Objective:** This study examined the willingness to get vaccinated and the factors influencing this attitude in extreme settings—in the Czech Republic (at the time of the survey, the third-worst affected country in the world).

**Methods:** We used national data from the general adult Czech population (N = 1,401) and measured attitudes towards vaccination, sociodemographic characteristics, government trust, knowledge about COVID-19 vaccines, personal characteristics, depression and anxiety.

**Results:** Respondents who were more likely to refuse the vaccine were: female, younger, living without a partner, self-employed or unemployed, living in a town, believers outside the church, and did not trust the government, obtained information about the vaccine from social media, were extroverts and depressed. Conversely, respondents who were less likely to refuse the vaccine were: pensioners, people with higher education, respondents with better real knowledge about the COVID-19 vaccines, those who obtained information about the vaccine from an expert and those who had higher scores in neuroticism.

**Conclusion:** This study thus offers a deeper understanding of the factors that might influence vaccine intention and subsequently the course of the COVID-19 pandemic.

## Introduction

Since December 2019, when Wuhan, China, became the centre of an outbreak of the disease COVID-19 (1), which subsequently spread around the world, the most interest has been focused on developing effective treatments of COVID-19 or developing safe and effective vaccines that are essential for the effective management of the pandemic (2).

In April 2021 (the time of our study), four vaccines had undergone phase 3 of a clinical trial; their efficacy had been proved and they were authorised for use in the European Union by the European Medicines Agency (EMA) (3). However, the mere presence of approved vaccines is not sufficient. So-called herd immunity is also needed to get the pandemic successfully under control. Based on the currently available epidemiological data, for a vaccine with a declared 90%–95% efficacy, the required herd immunity level would be 63%–76% (4, 5). To reach this limit, widespread vaccine acceptance and a willingness to be vaccinated are needed. However, studies on COVID-19 vaccination acceptance suggest that low vaccination rates have remained an issue of concern, while vaccine hesitancy and refusal are increasing (6, 7). In general, low willingness to get vaccinated is a growing problem, as also acknowledged by the WHO, which in 2019 (prior to the COVID-19 pandemic) labelled vaccine hesitancy among the top 10 threats to global health (8). Thus, there is a need to investigate what influences the willingness to be vaccinated. In the future, another situations similar to the current pandemic may arise and the willingness to be vaccinated may be essential in dealing with them.

Studies on COVID-19 vaccination hesitancy or refusal have shown that the common reason for COVID-19 hesitation or rejection is the fear of vaccine safety (9, 10) or its side effects, the belief that vaccines are not necessary (10), are effective (9–11), perceived unknown/short duration of immunity (11), or refusal of the vaccination generally (10). However, many factors can affect vaccination attitudes and can be complicated by the multifaceted nature of this phenomenon. Factors influencing the hesitation in accepting the vaccination could include cultural, sociodemographic, political, cognitive, psychological and spiritual factors (12–16). Thus, analysis of such factors in connection with COVID-19 vaccination is needed for guiding interventional measures for minimising the group of people refusing COVID-19 vaccines.

In connection with COVID-19 vaccination, research has shown that hesitation/refusing is associated, for example, with female gender, younger age, lower education (11, 17–20), living in a partnership (19), rural housing (11, 20), lower household income (11, 18–20), better health condition (17, 21), spirituality (22), religiosity (23, 24), belief in conspiracy theories (19, 25, 26) and poorer knowledge about COVID-19 (27). But to the best of our knowledge, this is one of the first studies that assesses the relationship between knowledge about COVID-19 vaccines with a tendency to refuse a COVID-19 vaccine, and in this relationship to compare the declared knowledge against the real knowledge.

Moreover, in April 2021 (the survey time), the Czech Republic (CZ) was the most affected country in Europe in terms of the number of positive cases per million inhabitants and the third-worst affected country in the world (28). CZ is also a country where vaccination is free for all, so there are no financial barriers affecting the willingness to be vaccinated. Therefore, it represents an interesting research area for examining the willingness to get vaccinated and the factors influencing this attitude. Thus, this study aims: 1) to examine the prevalence of people willing/refusing a COVID-19 vaccine in CZ and 2) to determine which of the assessed factors (sociodemographic, government trust, knowledge about COVID-19 vaccines, personal characteristics, anxiety, or depression) are related to the unwillingness to get vaccinated in such an “extreme” environment.

## Methods

### Participants and Procedure

For this study, we used data from an anonymous self-reported online survey gathered in CZ during the COVID-19 vaccination campaign in April 2021, when the current vaccination rate was around 10% (29). Based on power analysis and standard data collection practices in the Czech Republic, the minimum sample size was set at 1,000 respondents. To ensure a given number of quality respondent (high quality data), and given that the questionnaire was part of a larger survey covering also other topics, the aim was to collect data from 1,600 respondents. To achieve a balanced sample close to the adult Czech representative sample regarding gender and age, a professional was hired to collect the data. The final sample provided by the agency was 1,662 participants. However, 91 participants did not finish the survey (participants could only proceed to the next question after completing the current one), and thus only 1,571 participants remained. Subsequently, to ensure the high quality of the data, we identified and excluded respondents with 1) a unified pattern of responses and/or 2) extremely short time filling in the survey and/or 3) responding inconsistently to two control sets of questions regarding years, weight and height.

At the beginning of the survey, participants were made familiar with the aim and content of the survey, their rights and the handling of the data, and they had to explicitly express their agreement with each of the key points of the informed consent. Respondents also had to declare their willingness to participate in the survey by clicking on an appropriate button. Participation in the survey was entirely voluntary, and respondents could leave the survey at any time without giving a reason. A compensation for the study participation was secured by the professional agency according to their internal rules. Before the main study, a pilot study was realized among volunteers at home university. The study design was approved by the Ethics Committee.

### Measures

*COVID-19 vaccination intentions* were measured by the question: “Will you be or have you already been vaccinated with a currently available COVID-19 vaccine?” with possible answers: 1) “No,” 2) “I do not know yet,” and 3) “Yes.” For the purposes of further analysis, the responses were dichotomised; “no” was classified as vaccine refusal, and the response “I do not know yet” and “yes” formed the second group.

*Declared knowledge* was assessed by the question: “To what extent do you understand the principle of operation of COVID-19 vaccines?” Participants answered on a five-point scale ranging from “Not at all” (1; corresponding to no knowledge) to “Completely” (5; corresponding to excellent knowledge).

*Real knowledge* was measured by a knowledge test consisting of five questions about the COVID-19 vaccines. The questions were created based on well-known and publicly available information on the website of the State Institute for Drug Control (30). For example, one of the questions was: “RNA from the vaccine:” with possible answer 1) “remains in the body for a long time and still activates the production of B-lymphocytes needed for long-term immunity of the organism”; 2) “is decomposed shortly after vaccination”; and 3) “I do not know.” There was a possibility to answer “I do not know” for each question. One point was awarded to the respondent for each correct answer, and the incorrect answer or the answer “I do not know” was given 0 points. For the analysis, we used the total score (from all questions) as a continuous variable. A higher score corresponded to better knowledge.

#### Source of Information

A 10-item measure (a list of ten different possible sources of information) was used, introduced by the question: “Where do you get information about COVID-19 vaccines, from which you then decide whether to get vaccinated?” The sources are listed in Table 3.

*Verification of sources* was assessed by a question: “Do you verify the sources of information regarding vaccination?” with possible answers: 1) “always,” 2) “mostly yes,” 3) “rather not,” 4) “never.” Consequently, when the respondent marked “always” 1) or “mostly yes” 2), the respondent was classified as verifying.

*Religiosity* was assessed using the following question: “At present, would you call yourself a believer?” Possible answers were: 1) “Yes, I am a member of a church or religious society” (coded as “believer”); 2) “Yes, but I am not a member of a church or religious society” (coded as “believer outside the church”); 3) “No” (coded as “non-believer”); 4) “No, I am a convinced atheist” (coded as “atheist”).

*Government trust and the trust that the government is managing the pandemic* were measured by the question: “Do you currently have confidence in the government?” and “Do you feel that the government manages the current situation?” with possible answers 1) “definitely not”; 2) “rather not”; 3) “neither not, nor yes”; 4) “rather yes”; 5) “yes.” Subsequently, participants who answered “definitely not” and “rather not” (answers 1 and 2) were considered as “distrust” or “is not managing the pandemic,” respectively, and participants who answered with answers 3 to 5 as “trust or do not know” and “is managing the pandemic or do not know,” respectively.

*Personal characteristics* were measured using the Big Five Inventory (BFI), a self-reported questionnaire that assesses five personality domains, namely, openness, conscientiousness, extraversion, agreeableness, and neuroticism. Each item is evaluated on a five-point Likert scale ranging from “strongly disagree” (1) to “strongly agree” (5) (31). A 44-item version of the scale validated for the Czech environment (32) was used in the present study. Higher scores within the domains correspond with a greater propensity for the personality trait being measured. For the analysis, we used the total score of each domain as a continuous variable. Cronbach’s alpha was 0.82 for openness, 0.81 for conscientiousness, 0.83 for extraversion, 0.72 for agreeableness, and 0.86 for neuroticism.

*Anxiety* was measured using the abbreviated version of the Overall Anxiety Severity and Impairment Scale (OASIS), a short 5-item self-report tool (33) validated for the Czech environment (34). Its items measure the frequency and severity of anxiety symptoms. Respondents have to choose one of five responses that best illustrated their experience over the past week. The responses scale ranging from “Never” (0) to “Constantly/Extreme/All the Time” (4). For further analysis, we used the total score, which ranges from 0 to 20, as a continuous variable. A higher score suggested a higher level of anxiety. Cronbach’s alpha was 0.91 in our sample.

*Depression* was assessed using the Overall Depression Severity and Impairment Scale (ODSIS), a short 5-item self-report measure (35) validated for the Czech environment (34). The ODSIS is a tool used to assess the severity and frequency of depressive symptoms. The respondents answered, and responses were interpreted the same way as in the case of the OASIS. Cronbach’s alpha was 0.94 in our sample.

### Statistical Analyses

First, we performed descriptive analyses of the study sample. We then evaluated the attitudes towards COVID-19 vaccination. In the next step, the normality of the data was verified using the Shapiro-Wilk test. Because of the non-normal distribution of data, we assessed the associations between the COVID-19 vaccination refusal and sociodemographic variables (e.g., age, gender, economic status, faith) using separate-univariate binary logistic regression models, both crude and adjusted for gender, age and education. Using the binary logistic regression model avoids the normality assumption and offers an easier interpretation of the results. In the same way, we assessed the relation of COVID-19 vaccination refusal with government trust, declared and real knowledge of the COVID-19 vaccines, source of information about the COVID-19 vaccines, verification of information and personal characteristics. All analyses were performed using the statistical software package IBM SPSS version 25 (IBM Corp, New York, United States).

## Results

The sociodemographic characteristics of the sample are presented in Table 1. The final sample consisted of 1,401 participants (mean age = 50.46; SD = 15.73; 55.7% male). Most of the respondents (58.9%) reported COVID-19 vaccine acceptance. Further, 19.2% of respondent trusted the government, 16.3% were undecided and 20.9% of respondent trusted that government is managing the pandemic and 16.8% did not know.

**TABLE 1:** Description of the study sample (Czech Republic, 2021).

	Total		COVID-19 vaccine intentions					
			Acceptance		Hesitancy		Refusal	
	N	%	N	%	N	%	N	%
Gender								
Male	781	55.7	481	61.6	172	22.0	128	16.4
Female	620	44.3	344	55.5	142	22.9	134	21.6
Age								
15–34	116	8.3	57	50.0	31	21.4	28	28.6
35–49	445	31.8	205	50.7	129	25.4	111	23.9
50–65	367	26.2	367	64.6	208	21.1	80	14.3
66–99	473	33.8	355	66.3	74	19.8	44	13.9
Family status								
Married/partnership	894	63.8	553	61.9	193	21.6	148	16.6
Single/divorced/widow (er)	507	36.2	272	53.6	121	23.9	114	22.5
Economic status								
Student	50	3.6	26	52.0	12	24.0	12	24.0
Employee	682	48.7	373	54.7	171	25.1	138	20.2
Self-employed	88	6.3	51	58.0	16	18.2	21	23.9
Unemployed	61	4.4	21	34.4	18	29.5	22	36.1
Disabled/old-age pensioner	465	33.2	337	72.5	76	16.3	52	11.2
Household/maternity leave	55	3.9	17	30.9	21	38.2	17	30.9
Education level								
Elementary	84	6.0	42	50.0	18	21.4	24	28.6
Secondary vocational	531	37.9	269	50.7	135	25.4	127	23.9
Secondary graduation	427	30.5	276	64.6	90	21.1	61	14.3
College	359	25.6	238	66.3	71	19.8	50	13.9
Living place								
Town	961	68.6	558	58.1	207	21.5	196	20.4
Village	440	31.4	267	60.7	107	24.3	66	15.0
Religiosity								
Religiously affiliated	131	9.4	80	61.1	28	21.4	23	17.6
Religiously non-affiliated	329	23.5	177	53.8	75	22.8	77	23.4
Non-religious	680	48.5	400	58.8	155	22.8	125	18.4
Convinced atheist	261	18.6	168	64.4	56	21.5	37	14.2
Government trust								
Trust/I do not know	498	35.5	354	71.1	95	19.1	49	9.8
Distrust	903	64.5	471	52.2	219	24.3	213	23.6
Government manages pandemic								
Is managing/I do not know	528	37.7	363	68.8	106	20.1	59	11.2
Is not managing the pandemic	873	62.3	462	52.9	208	23.8	203	23.3
Total	1,401	100	825	58.9	314	22.4	262	18.7

Regarding the personal characteristics, the respondents achieved the following mean scores: openness 32.96 (SD = 5.79), conscientiousness 31.25 (SD = 4.91), extraversion 24.29 (SD = 5.09), agreeableness 31.46 (SD = 4.48), neuroticism 22.67 (SD = 5.87), OASIS 9.35 (SD = 4.18), ODSIS 8.69 (SD = 4.33). Regarding the knowledge the mean value of the declared knowledge was 3.36 (SD = 1.12), which corresponds roughly to intermediate knowledge. The mean value of the real knowledge was 1.02 (SD = 1.24), which means that, on average, respondents answered only one question correctly. So the real knowledge was much lower than the declared knowledge.

Table 2 shows the associations of selected factors COVID-19 vaccination refusal. A significantly higher tendency toward vaccination refusal was observed among women, people living without a partner, self-employed people (adjusted model), unemployed people (adjusted model), respondents living in a town, believers outside the church and also respondents who did not trust the government or those who thought that government was not managing the pandemic.

**TABLE 2:** Association of sociodemographic characteristics, religiosity and government trust with COVID-19 vaccine refusal: results of binary logistic regression, crude and adjusted for age, gender and education level, leading to odds ratios with 95% confidence intervals (Czech Republic, 2021).

	Vaccine refusal crude	Vaccine refusal adjusted
Gender		
Male	1	1
Female	1.41 (1.08–1.84)*	1.32 (1.00–1.74)*
Age	0.97 (0.96–0.98)***	0.97 (0.96–0.98)***
Family status		
Married/partnership	1	1
Single/divorced/widow (er)	1.46 (1.11–1.92)**	1.45 (1.09–1.92)*
Economic status		
Student	1	1
Employee	0.80 (0.41–1.58)	1.93 (0.86–4.32)
Self-employed	0.99 (0.44–2.24)	2.77 (1.09–7.05)*
Unemployed	1.79 (0.78–4.11)	3.58 (1.82–9.27)**
Disabled/old-age pensioner	0.40 (0.20–0.81)*	1.76 (0.66–4.68)
Household/maternity leave	1.42 (0.60–3.37)	1.87 (0.73–4.82)
Education level		
Elementary	1	1
Secondary vocational	0.79 (0.47–1.31)	1.13 (0.66–1.94)
Secondary graduation	0.42 (0.24–0.72)**	0.66 (0.37–1.16)
College	0.41 (0.23–0.71)**	0.55 (0.31–0.98)*
Living place		
Village	1	1
Town	1.45 (1.07–1.97)*	1.42 (1.04–1.94)*
Religiosity		
Believer	1.29 (0.73–2.28)	1.32 (0.73–2.37)
Believer outside the church	1.85 (1.20–2.85)**	1.68 (1.07–2.61)*
Non-believer	1.36 (0.92–2.03)	1.18 (0.78–1.77)
Convinced atheist	1	1
Government trust		
Trust/do not know	1	1
Distrust	2.83 (2.03–3.94)***	2.35 (1.66–3.35)***
Government is managing the pandemic		
Manages the pandemic/I do not know	1	1
Does not manage the pandemic	2.41 (1.76–3.29)***	2.02 (1.46–2.81)***

Notes: *p < 0.05, **p < 0.01, ***p < 0.001; *p*-values below 0.05 are considered significant and are shown in bold.

In contrast, a lower tendency to vaccination refusal was significantly associated with age, with a 3% decrease in the odds ratio (*p* ˂ 0.001) for each year. Also, disabled/old pensioners (crude model) and respondents with a higher education (crude model) were less likely to refuse vaccination.

Table 3 shows the associations of COVID-19 vaccine refusal with COVID-19 knowledge, information source and information verification. Respondents’ declared knowledge (crude and adjusted models) and their real knowledge (crude models) about the COVID-19 vaccines were associated with a decreased tendency towards vaccination refusal, with associations ranging from OR = 0.79 (*p*˂0.001) for declared knowledge to OR = 0.87 (*p*˂0.05) for real knowledge (crude models). Regarding the source of information, in the crude model on real knowledge, respondents who obtained information about vaccination from experts (doctor or other experts) were 25% less likely to refuse a COVID-19 vaccine, and on declared knowledge, respondents who obtained information about vaccination from social sources (e.g., friends, social media or e-mail) were 1.45-times more likely to refuse a COVID-19 vaccine (*p* ˂ 0.05). Verification of information did not significantly affect the willingness to be vaccinated.

**TABLE 3:** Association of declared and real knowledge, source of information and verification of information sources with refusal of COVID-19 vaccination: results of binary logistic regression crude and adjusted for age, gender and education level, leading to odds ratios with 95% confidence intervals (Czech Republic, 2021).

	Vaccine refusal	
	Crude	Adjusted
Knowledge about COVID-19 vaccines		
Declared	0.79 (0.70–0.89)***	0.85 (0.75–0.96)**
Real	0.87 (0.77–0.97)*	0.90 (0.80–1.02)
Source of information		
Official and scientific	1.19 (0.91–1.57)	1.28 (0.96–1.71)
Experts	0.75 (0.57–0.98)*	0.80 (0.60–1.06)
Social media	1.45 (1.09–1.91)*	1.28 (0.96–1.71)
Internet and TV/radio	0.97 (0.74–1.27)	1.18 (0.89–1.58)
Verification of information		
Not verifying	1	1
Verifying	0.81 (0.61–1.07)	0.92 (0.69–1.22)

Notes: *p < 0.05, **p < 0.01, ***p < 0.001; *p*-values below 0.05 are considered significant and are shown in bold.

Official and scientific sources—State Institute for Drug Control, Ministry of Health, vaccine package leaflet scientific articles; Experts—Doctor, other experts; Social media—friends/acquaintances/family, social media, e-mail; Internet and TV/radio—internet (other sources than the above options), TV/radio.

Table 4 shows associations of COVID-19 vaccine refusal with personal characteristics, anxiety and depression. Regarding personal characteristics, an increased extroversion score was significantly associated with a higher probability of COVID-19 vaccine refusal, with a 17% (crude) or 16% (adjusted) increase in the odds ratios (*p* ˂ 0.05) for each point of the extroversion score. Furthermore, a higher level of neuroticism was related to a lower chance of refusing vaccination, with a 17% decrease in the odds ratios (*p* ˂ 0.01, adjusted). Moreover, the table reveals that respondents’ higher level of depression was connected with a higher tendency to refuse the vaccination. Other associations were not significant.

**TABLE 4:** Association of personal characteristics (standardised to z-scores) with attitudes towards refusing COVID-19 vaccination: results of binary logistic regression, crude and adjusted for age, gender and education level, leading to odds ratios with 95% confidence intervals (Czech Republic, 2021).

	Vaccine refusal crude	Vaccine refusal adjusted
BFI		
Openness	1.08 (0.94–1.24)	1.15 (1.00–1.32)
Conscientiousness	0.94 (0.82–1.08)	1.00 (0.85–1.12)
Extraversion	1.17 (1.02–1.35)*	1.16 (1.01–1.33)*
Agreeableness	0.96 (0.83–1.10)	0.99 (0.86–1.14)
Neuroticism	0.96 (0.84–1.10)	0.83 (0.72–0.95)**
OASIS	1.12 (0.98–1.28)	1.00 (0.87–1.15)
ODSIS	1.15 (1.01–1.31)*	1.03 (0.89–1.18)

Notes: *p < 0.05, **p < 0.01; *p*-values below 0.05 are considered significant and are shown in bold.

## Discussion

This study aimed to examine the prevalence of people accepting/refusing a COVID-19 vaccine and to determine which factors affect the unwillingness to get vaccinated in the Czech Republic, one of the countries most affected by COVID-19 infection in the world (28). The results showed that only 59% of respondents were willing to get vaccinated. Respondents who were more likely to reject the vaccine were: female, younger, living without a partner, self-employed and unemployed, living in a town, believers outside a church, and who did not trust the government or did not trust that the government is managing the pandemic, who obtained information about the vaccine from social media, were extroverts and those who felt depressed. In contrast, respondents who were less likely to refuse the vaccine were: disabled or old-age pensioners, people with higher education, respondents with better knowledge of the COVID-19 vaccines, who obtained information about vaccines from experts and who had higher scores in neuroticism.

We found that 58.9% of respondents reported COVID-19 vaccine acceptance, 18.7% vaccine refusal and 22.4% hesitancy. The worldwide willingness to be vaccinated was estimated in a meta-analysis by Nehal et al. (36) at 66% and the European willingness was 67%. At the time of the study, CZ was the third-worst affected country in the world (28). Given that a higher risk of infection is associated with a higher willingness to be vaccinated (21), it would be expected that in this situation the willingness to be vaccinated would be higher in CZ. However, CZ is below European and global estimates. Although some sociodemographic characteristics influence the willingness to be vaccinated, as described below, there are also other factors that could reduce the willingness to be vaccinated in comparison to other countries. Some of these possible factors are presented in the second part of the Discussion.

Our second observation was that females and younger people were more likely to refuse a COVID-19 vaccine, which is in line with majority of other studies (11, 17–20, 23, 37–39), however, a minority of studies where men were more likely to refuse a COVID-19 vaccine (40, 41). Women and younger people do not belong to the risk group for severe and critically ill COVID-19 patients (42), so they may not have felt as threatened. In contrast, pensioners belong to the risk group (due to their age) and we observed, like Freeman et al. (19), a higher willingness to be vaccinated. Consequently, we also found that people with higher education were less likely to refuse a COVID-19 vaccine which is in line with other studies (11, 17–19, 23). Furthermore, similar to other studies that have found religious people to be less willing to be vaccinated (23, 24), our study also shows that religiosity had an influence on vaccine refusal but only in case of believers outside the church. It is possible that believers are more influenced by the official position of the Roman-Catholic Church (the most widespread church in the Czech Republic), which recommended vaccination (43). It is thus possible that not religiosity itself but other factors (e.g., spirituality, especially some of its forms highlighting one’s inner power and individuality) are a barrier to vaccine uptake (22).

In our study, another group refusing COVID-19 vaccination comprised people living without a partner. The opposite findings were reported by Freeman et al. (19) and Giuseppe et. Al (37). Further, other studies also came to different conclusions regarding vaccine refusal among the self-employed or unemployed (40, 41, 44) and people living in urban areas (11, 20). These discrepancies may be caused by various factors. First, when comparing the willingness/unwillingness to be vaccinated and its association with sociodemographic factors, the fact that different studies had various sample compositions (e.g., regarding age, gender, education) and used different designs should be taken into consideration. Hence, comparisons are only possible to a limited extent. Second, determining the sociodemographic factors of acceptability of vaccines is complex and context-specific (e.g., demographic or historical differences).

Furthermore, we found that, in general, only 19.2% of people trusted the government, and 20.9% believed the government was handling the COVID-19 pandemic. The proportion of people who trusted the government is significantly lower compared to other countries (e.g., Australia 80% and New Zealand 83%) (45). This is also confirmed by OECD data from 2020 (46), where CZ was among seven countries with the lowest level of trust in government. Moreover, one of the strongest relationships with vaccine refusal was distrust in the government or its managing of the pandemic. These observations align with the findings of other authors who mention that lower government trust and the measures they implemented during the COVID-19 pandemic were associated with delaying or refusing a COVID-19 vaccine (21, 47). Thus, low trust in the government could explain the low levels of vaccination uptake in CZ.

Further, we found that there were differences between declared knowledge about the COVID-19 vaccines and real knowledge (as assessed by control questions), and that real knowledge was lower than declared knowledge. This phenomenon is entirely consistent with the Dunning-Kruger effect, which describes that people tend to have an overly favourable opinion of their own abilities in many social and intellectual domains (48). However, though real knowledge was associated with a higher willingness to be vaccinated, the same held for declared knowledge, which corresponds to the findings of other authors (20, 49).

Although, as mentioned above, it does not seem to matter whether knowledge is real or claimed, our data suggest that the sources of information matter. Participants drawing information from experts were more likely to accept vaccines, which is consistent with other studies that have shown the important role of the healthcare workers as source of information with a positive impact on willingness to be vaccinated (50, 51). In contrast, participants drawing information from social media were more likely to refuse vaccines. Similar findings were observed by Reno et al. (52), who suggested that social media directly or indirectly increase vaccine hesitancy towards COVID-19 vaccination, while the opposite effect was observed for institutional websites. An explanation for this could be that social media have been identified as major vectors for the dissemination of misinformation and conspiracy theories (53–55), and at the same time, people who believe in COVID-19 conspiracy theories are less willing to get vaccinated (25, 26).

Further, our data also suggest that respondents with a higher score for extroversion had a higher probability of vaccine refusal. Conversely, people with a higher score for neuroticism had a lower likelihood of vaccine refusal. In contrast, Howard’s study (56) suggested that extroversion has positive effects on pro-vaccine outcomes and explained this by the fact that extroversion is associated with positive expectations (57), so they could have expected greater benefits from vaccination (56). However, this positivity can also be turned towards a positive expectation of a disease course (mild course) and an associated lower need to be vaccinated. In addition, extroverts are also likely to use social media (58). As we mentioned in the previous paragraph, social media could lead to less willingness to be vaccinated. Regarding neuroticism, the explanation could be that neuroticism is positively related to a perceived risk of infection (59), which may lead these respondents to be more willing to get vaccinated for their own safety.

Finally, we found that with depression, the chance of vaccine refusal also increased, while anxiety did not affect attitude toward vaccination. These findings are not consistent with other studies, which are, however, heterogenous themselves. Urrunaga-Pastor et al. (60) found that having depressive symptoms was associated with a higher probability of vaccine intention, but Bendau et al. (61) did not find any relationship. This inconsistency is probably due to different ways of measuring depression. Thus, it is not easy to interpret the findings, and this relationship needs to be verified in subsequent studies on a larger sample. With regards to anxiety, we found no significant association, which is consistent with study by Bendau et al. (61).

### Strengths and Limitations

The strength of this study is that it captures the willingness to be vaccinated and the factors influencing the unwillingness to be vaccinated in the country (CZ), which at the time of the survey was the worst affected country in Europe and the third-worst affected country in the world. To the best of our knowledge, this is one of the first studies examining the association of attitudes towards vaccination and personal characteristics, and assessing the relationship between knowledge about COVID-19 vaccines with a tendency to refuse COVID-19 vaccines. A further strength is that we changed the order of the questions in the questionnaire, which allowed us to eliminate the order effect. Another strength is the large sample close to national sample characteristics regarding age and gender.

However, this study also has some limitations. The first limitation could be social-desirability bias, because our study used data based on self-reports of the participants. Second, although the sample is close to national sample characteristics, our study also suffers from sampling bias, as the data collection was done online. Therefore, surveys were limited to internet users and users interested or willing to participate in online studies. Thus, vulnerable populations are likely underrepresented. For this reason, our findings cannot be generalized to the general population. Third, given that our independent–dependent variable relationship could be distorted by some other unknown factor, confounding bias could also be present. Finally, our cross-sectional design does not allow us to conclude causality.

### Implications

Our findings suggest that attitudes toward vaccines could be affected through several factors, including sociodemographic characteristics, personal characteristics, knowledge about vaccines and government trust. The lack of trust in the government could be one of the significant factors behind the overall below-average willingness to be vaccinated in the Czech Republic (compared to European or global estimates of willingness to be vaccinated). In this study, we highlight important predictors that may influence vaccination decisions. This can be very useful for all those working on vaccination campaigns to prevent the spread of the current coronavirus, but also the knowledge gained in this COVID-19 pandemic is highly valuable for managing possible future pandemics or more generally for communication strategies for different vaccinations in the future.

Further research should focus on the causal effects of individual factors on the attitudes towards vaccination. It could also be helpful to focus on the other specific factors of vaccine refusal apart from those mentioned in this study.

### Conclusion

Our findings suggest that the Czech Republic had a below-average willingness to be vaccinated, even though it was strongly affected by the COVID-19 pandemic. Among factors associated with an attitude toward vaccine acceptance were sociodemographic factors, trust in the government, knowledge about COVID-19 vaccines, information source, personal characteristics and depression. Thus, this study offers a deeper understanding of the factors that might influence vaccine intention and, subsequently, the managing of COVID-19 pandemic.
